# Global Inequities in Unpaid Cancer Caregiving: A Systematic Review and Exploratory Meta‐Analysis of Time and Cost Burden

**DOI:** 10.1002/cam4.71657

**Published:** 2026-02-20

**Authors:** Lan Gao, Neha Das, Shalika Bohingamu Mudiyanselage, Natalie Winter, Stephanie Cowdery, Victoria White, Patricia M. Livingston

**Affiliations:** ^1^ Deakin Health Economics, School of Health & Social Development, Institute for Health Transformation Deakin University Geelong Victoria Australia; ^2^ Centre for Quality and Patient Safety Research, Institute for Health Transformation, School of Nursing and Midwifery Deakin University Geelong Victoria Australia; ^3^ School of Psychology, Faculty of Health Deakin University Geelong Victoria Australia; ^4^ Faculty of Health Deakin University Geelong Victoria Australia

**Keywords:** Cancer, informal care, socio‐demographic index

## Abstract

**Background:**

Unpaid caregiving is a critical but often under‐recognised component of the cancer care continuum. As cancer prevalence rises globally, particularly in lower‐income countries, quantifying the time and economic burden of unpaid caregiving across regions is essential for health resource planning, economic participation and support services. This systematic review aimed to quantify the global burden of unpaid cancer caring by examining care hours and associated costs, disaggregated by country income level based on the Socio‐Demographic Index (SDI).

**Methods:**

We systematically searched databases for studies published up to January 2026 that reported unpaid cancer caregiving hours and/or costs. Data were extracted and synthesised narratively, and where appropriate, pooled using random‐effects meta‐analysis for unpaid care hours. Study quality was assessed using standard checklists appropriate to the study design.

**Results:**

Twenty‐six studies met the inclusion criteria, with the majority conducted in high‐income countries. Unpaid care hours ranged from 1.3 to 113.8 h per week, with a pooled estimate of 48.35 h (95% CI: 17.81–78.89), though heterogeneity was high (*I* ^2^ = 100%). Unpaid care costs varied widely, with monthly costs averaging US$2249 (range: US$346–US$5626) in high‐SDI countries; US$196 in high‐middle SDI countries; US$26 in middle SDI countries; US$24 in low‐middle SDI countries; and US$37 in low‐SDI countries. Costing methods varied, with opportunity cost and replacement cost approaches most commonly used.

**Conclusions:**

Unpaid carers dedicate substantial time to support people living with and beyond cancer, offering essential assistance that fills critical gaps in health services. This contribution represents a substantial economic burden that remains largely uncompensated. There is a striking lack of data from low‐ and middle‐income countries, where the incidence of cancer is rising, and unpaid care resources are limited. Future research must prioritise recognition, valuation and support for unpaid carers, thus helping increase economic productivity, particularly in underserved settings.

## 1 Introduction

The global cancer burden is increasing steadily due to increasing exposure to risk factors, including tobacco, dietary fat consumption, alcohol and air pollution [1], ageing, population growth and advances in screening and detection [1]. The burden is particularly pronounced in low‐income countries, where access to timely and high‐quality cancer care is often constrained by limited resources, diagnostic delay and competing health and economic consequences due to socioeconomic disadvantages compared to developed countries [2, 3].

Global Cancer Statistics show that over 20 million people were newly diagnosed with cancer in 2022 [4]. This number is expected to rise to 35 million by 2050, representing a 77% increase from 2022 [4]. Data from the Global Burden of Disease (GBD) study (2021) further reveal substantial disparities in cancer epidemiology across countries of different economic development [5]. While in 2019 the largest absolute numbers of cases and deaths occurred in the higher Socio Demographic Index (SDI) quintiles (10,900 per 1000 incidence in high SDI and 132.7 per 1000 deaths in high middle SDI), the largest increasing annualised rates of change in the absolute numbers of cases and deaths from 2010 to 2019 occurred in the low‐middle and low SDI quintiles (0.9% and 0.3%, respectively) [5]. This trend indicates that by 2040, more than two‐thirds of the world's cancers will occur in low‐income and middle‐income countries [5].

As health care systems face growing pressure to manage cancer care across the disease trajectory, from diagnosis through survivorship to end of life, unpaid caring has become an indispensable component of cancer care [6]. Unpaid carers, such as partners, close family members, or friends, offer essential physical, emotional, social and financial support, often without any form of financial compensation [7]. Caregiving is a heterogeneous concept, as it is impacted by multiple factors such as the caregiver's capacity to perform caregiving activities, coping strategies, competing commitments and availability of paid domestic helpers [8]. Caring tasks can include supporting someone through diagnostic and treatment procedures, medication management, emotional support, daily living assistance and, in many cases, palliative care [6, 7, 9]. The average length of time cancer carers provide unpaid care for a person with cancer ranges from 6 months to 2 years [10], though some carers provide support over even longer periods [11]. As health systems have shifted cancer care from inpatient to outpatient and home settings, the demand for unpaid caregiving will continue to grow [12]. This has major implications not only for carers' health and wellbeing but also for economic productivity and healthcare resource planning [6, 12]. However, despite the critical role of unpaid caregiving, there is limited global evidence quantifying its time and economic burden, particularly across countries with differing income levels. Understanding how the caregiving burden varies by national income is essential to inform equitable health system responses, social protection policies and carer support strategies. A previous scoping review exploring the indirect cost of cancer included studies up to 2018 [13]. They reported average out of pocket costs of CA$447, workplace absenteeism costs of CA$207 and unpaid care costs of CA$4809 (replacement cost approach) or CA$2877 (opportunity cost approach) [13]. The authors highlighted the lack of literature in low and middle income countries as a significant gap in research conducted to date [13]. However, the review did not examine the volume of unpaid care provided—such as the number of caregiving hours—across the included studies.

Building on Coumoundouros et al.'s [13] prior work, we conducted a systematic review to quantify the burden of unpaid caregiving for cancer by country, classified into five SDI levels. We also performed a meta‐analysis of unpaid care hours, providing insights into the magnitude of this often‐overlooked but vital component across the cancer care continuum.

## 2 Methodology

This systematic review followed the (Preferred Reporting Items for Systematic reviews and Meta‐Analyses (PRISMA)) reporting guidelines [14], and the PROSPERO registry number was 2025 CRD420251028229.

### 2.1 Search Strategy

We searched key databases (MEDLINE, Global Health, CINAHL via EBSCO, EMBASE) to identify articles meeting our eligibility criteria. The search spanned articles from January 2015 to April 2025 in the first screening exercise. To provide an updated review, a second screening exercise was conducted in January 2026, which included searches from April 2025. The search terms included cancer, unpaid caregiving and time or cost. The search strategy and results from MEDLINE are included in Table S1.

### 2.2 Eligibility Criteria

#### 2.2.1 Study Design

Original research studies reporting data on cancer unpaid caregiving time and/or costs were eligible. As such, the inclusion criteria are reported as follows:

- Participants: Eligible studies focused on unpaid carers of cancer patients, including adults and children.
- Intervention and comparator: Studies involving any intervention and comparator were eligible for inclusion.
- Outcomes: Studies focusing on the time or costs associated with unpaid cancer caregiving were included.
- Studies in English were included.
- Peer‐reviewed studies were included.

#### 2.2.2 Exclusion Criteria

Qualitative studies, clinical guidelines, conference abstracts and study protocols were excluded. Studies using methods such as model‐based predictions for unpaid caregiving time and cost, or those that did not provide information on the time or costs of cancer unpaid caregiving, were excluded. Studies in non‐English languages were excluded as the impact of non‐inclusion of these studies was determined to be minimal through a pilot check in MEDLINE.

### 2.3 Data Extraction, Management and Analysis

EndNote [15], Research Screener [16] and Covidence [17], platforms were used for screening the studies in the first screening exercise (January 2015 to April 2025). The review process involved two stages: title and abstract screening and full‐text screening. Two reviewers (ND, SBM) independently assessed the titles and abstracts of articles to determine eligibility for full‐text screening using Research Screener. Research Screener is a web application that utilises machine learning techniques to semi‐automate the title and abstract screening. The effectiveness and utility of the Research Screener have been affirmed in published systematic reviews [18, 19, 20, 21]. Researchers upload the total articles for title and abstract screening, and at least one seed article assessed as highly relevant in the Research Screener. The platform uses seed article(s) to identify relevant articles and provides 50 articles in the first iteration to the reviewers for screening. Based on the articles screened by independent reviewers in this iteration, the platform then provides the next list of 50 articles to the reviewers to be screened. This process continues as the platform provides 50 articles in every iteration and learns through the screened articles to identify relevant articles in the next screening round [16]. Reviewers can stop screening when no relevant articles are found.

All articles that potentially met the eligibility criteria were retrieved and evaluated by two reviewers for final inclusion through full‐text screening using Covidence. At the data extraction stage, two reviewers screened the included articles. A third reviewer resolved any disagreements (LG). Finally, the systematic reviews were also searched to identify any potential additional studies.

Data from the finalised studies were extracted in Microsoft Excel. The study characteristics (author, year of publication, country of origin, sample size), cancer characteristics (type, stage and time since diagnosis), characteristics of the person living with cancer and unpaid carer (age, gender and relationship), caregiving time (hours per day/year/other and total caregiving period so far, methods used to collect caregiving time), unpaid care cost (unit cost, method for calculating unpaid caregiving cost: opportunity cost approach, replacement cost approach, or other) were extracted.

Countries of origin for different studies were grouped into five categories based on the GBD's definition of SDI (range 0–1): low (0–0.47), low‐middle (0.47–0.62), middle (0.62–0.71), high‐middle (0.71–0.81) and high (0.81–1) [22, 23]. This categorisation is defined to analyse health outcomes and disease burden across different development levels [22].

### 2.4 Risk of Bias

Critical appraisal was determined using the Joanna Briggs Institute (JBI) Critical Appraisal Checklist for cross‐sectional and cohort studies [24] and Schnitzler et al. [25] was used to appraise the cost of illness studies. There are eight questions in the JBI checklist for cross‐sectional studies and ten for the cohort studies, and responses can be in the form of Yes, No, Unclear and Not applicable [24]. The response options in the checklist by Schnitzler et al. [25] include 17 items, and options include Yes, No, Partially, Not Applicable (NA) and Unclear.

### 2.5 Data Synthesis

The range of unpaid care hours, unit costs and total unpaid care costs across the studies by the GBD's SDI levels is reported. Additionally, the arithmetic means of unpaid care hours, unit costs and total costs were calculated by country SDI. As there was substantial heterogeneity in the method of reporting hours, certain calculations were undertaken. First, weekly unpaid care hours were calculated for the studies where they were reported in other denominations such as hours per day, per month, etc. Secondly, if studies reported hours by different stages of caring, a mean was calculated for that study. Finally, where hours were categorised and the percentage of carers devoting the categorised hours of caring was reported, the hours of caring for a majority of carers were used for the calculation of the mean. Followed by this, the mean scores across all studies were reported. Similar approaches were followed for unpaid care costs. Where studies reported multiple unit costs, the mean was calculated for that study; further, the mean across all studies was calculated and reported. In addition, where a study included multiple countries, data for individual countries were extracted and reported based on the country's SDI. Where applicable, monthly unpaid care costs were estimated by multiplying unpaid care hours by the corresponding unit cost for each SDI level. The unpaid care unit cost and total cost were converted to 2024 US$ values using purchasing power parity (PPP).

### 2.6 Exploratory Meta‐Analysis of Unpaid Care Hours

An exploratory meta‐analysis using a random effects model was conducted to calculate the pooled unpaid care hours across the studies. As such, the aim of the meta‐analysis was to explore the feasibility of quantitatively synthesising the cancer unpaid care hours and explain if an SDI level impacts unpaid care, that is, to search for the cause of this effect size variability [26]. Studies that reported sample size, mean and either standard deviation or 95% confidence intervals (CI) were included in the meta‐analysis. The analysis was conducted using STATA BE 18.0.

## 3 Results

**FIGURE 1:**
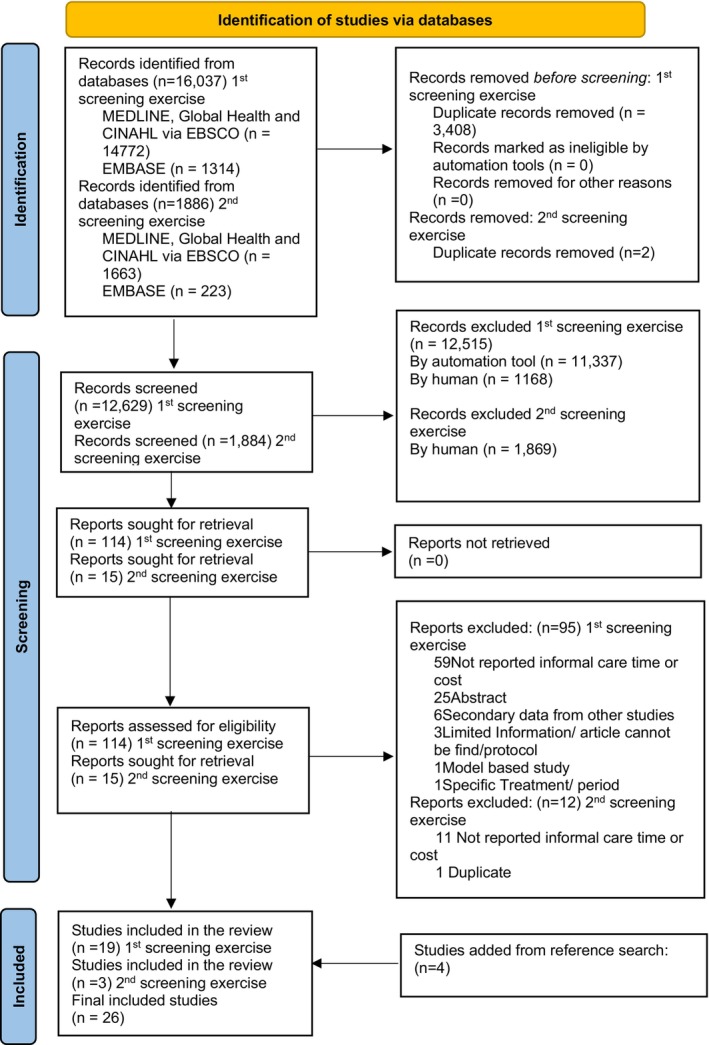
PRISMA flowchart.

A total of 16,037 studies were identified, and after removing 3408 duplicate articles, 12,629 were screened. A total of 114 studies underwent full‐text screening, with 19 studies meeting the inclusion criteria. An additional four studies were included through reference list review of previous systematic reviews, bringing the total eligible studies to 23. In the second round of review, where studies were searched from April 2025 to January 2026, 1884 unique articles were identified. Among these, 15 were searched for full‐text screening, and 3 were included in the final data analysis, bringing the total number of studies for inclusion to 26. These finalised studies reported data from separate datasets. The Preferred Reporting Items for Systematic Reviews and Meta‐Analyses (PRISMA) flowchart is shown in Figure 1.

Study characteristics are summarised in Table 1. The majority of the studies were conducted in high‐income countries, with one study from a high‐middle‐income country (Turkey) [49] one from a low‐middle‐income (Nigeria) [50] and two studies including countries across multiple SDI levels: one involved Australia, Canada, UK, USA, Colombia, Kazakhstan, Malaysia, India, Kenya, Nigeria, Malawi [51] and the other incorporated 27 multiple European Union countries [52]. The most frequently represented countries were Italy (*n* = 5) [30, 38, 41, 46, 52], USA (*n* = 4) [36, 37, 44, 45, 47, 51], Ireland (*n* = 4) [32, 33, 34, 52], UK (*n* = 3) [40, 51, 52], Finland (*n* = 3) [31, 39, 52], France (*n* = 3) [27, 46, 52] and Canada (*n* = 3) [28, 43, 51]. Most studies were cross‐sectional in design, with only three cohort [28, 30, 31] and five as cost‐of‐illness studies [35, 43, 49, 51, 52].

**TABLE 1:** Characteristics of included studies and informal carer details.

Sr no	Study details	Study country	SDI region	Data collection year	Time since diagnosis	Type of cancer	Stage of cancer	Mean age of cancer patient	Mean age of caregiver	Relation to cancer patient	Gender of caregiver
1	Bayen et al. [27]^b^	France	High SDI	2012–2013	Not reported	Glioma		Not reported	Not reported	Not reported	Not reported
2	Cai et al. [28]^c^	Canada	High SDI	2013–2017	Not reported		Palliative care period	76	59.15	Spouse 113 (43.13%) Children 124 (47.33%) Others 25 (9.5%)	Female 183 (69.85%) Male 79 (30.15%)
3	Chua et al. [29]^b^	Singapore	High SDI	Not reported	< 6 months, (31.3%) 6 months–1 year, (6.3%) 1–2 years, (12.5%) 2 years, (50%)	Breast 3 (18.8%) Gastrointestinal 6 (37.5%) Head and neck 1 (6.3%) Genitourinary 3 (18.8%) Gynaecological 1 (6.3%) Haematological 1 (6.3%) Respiratory (lung) 1 (6.3%)	Advance stages	63	43.8	Spouse 4 (25.0%) Child 7 (43.8%) Sibling 2 (12.5%) Extended family 3 (18.8%)	Male 7 (43.8%) Female 9 (56.3%)
4	Gridelli et al. [30]^c^	Italy	High SDI	2004	22.0 months	Lung cancer	III 18% IV 82%	65.5	54.7	Spouse 70% Son/daughter 18% Others 12%	Females 74%
5	Haltia et al. [31]^c^	Finland	High SDI	2009–2011	2.4 (0–15.2 years)	Breast cancer Colorectal cancer Prostate cancer	Palliative care period	69	Not reported	Not reported	Not reported
6	Hanly et al. [32]^b^	Ireland	High SDI	2008	Not reported	Colorectal cancer	Stage 1/2, 35.7% Stage 3/4, 50.0%	≤ 64, 6% 65–74, 28.6% ≥ 75, 25.3%	< 55, 33.8% 55–64, 29.9% ≥ 65, 33.8%	Spouse/partner, 72.7% Other, 27.3%	Male, 18.2% Female, 81.8%
7	Hanly et al. [33]^b^	Ireland	High SDI	Not reported	Not reported	Colorectal cancer	I/II: 35.7% III/IV‐50%		< 55, 52, 33.8% 55–64, 46, 29.9% > 65, 52, 33.8%	Spouse or cohabiting partner 112, 72.7% Othera 42, 27.3%	Male 28, 18.2% Female 126, 81.8%
8	Hanly et al. [34]^b^	Ireland	High SDI	2012	5.4 years	Head and Neck Cancer	Not reported	63	57.3	Not reported	Male 43, 24% Female 136, 76%
9	Hao et al. [35]^d^	Sweden	High SDI	2013	Not reported	Not reported	Not reported	Not reported	Not reported	Not reported	Not reported
10	Hendricks et al. [36]^b^	USA	High SDI	Not reported	Not reported	Not reported	Not reported	Not reported	16.13	Children	Male 17.3% Female 82.7%
11	Li et al. [37]^b^	USA	High SDI	Not reported	1 year	Prostate cancer	Not reported	< 65, 44, 50% 65+, 44, 50%	< 65, 60, 68,2% 65+, 28, 31.8%	Spouse/partner	Female 100%
12	Palandri et al. [38]^b^	Italy	High SDI	2012–2013	Not reported	Myelofibrosis	Not reported	Not reported	Not reported	Not reported	Not reported
13	Roine et al. [39]^b^	Finland	High SDI	2010	Not reported	Breast cancer	Not reported	All stages 61.7 (range 26–90)	Not reported	Not reported	Not reported
14	Rowland et al. [40]^b^	UK	High SDI	2015	Not reported	Lung, colorectal, prostate, breast, pancreas, oesophagus	Not reported		74.41	Spouse/partner 669 (44.5) Parent 650 (43.2) Someone else 157 (10.4)	Female 64.2%
15	Spatuzzi et al. [41]^b^	Italy	High SDI	2020	< 65: 647.8 days > 65: 898.2 days	All kinds	Not reported	< 65: 69.1 > 65: 75.0	< 65: 48.4 > 65: 72.9	< 65 years > 65 years Wife 4 (4.3) 14 (17.5) Husband 6 (6.4) 40 (50.0) Father 32 (34.0) 1 (1.3) Mother 35 (37.2) 5 (6.3) Son/daughter 0 (0.0) 3 (3.8) Other 17 (18.1) 17 (21.3)	< 65: 19%/81% > 65: 25%/75%
16	Stoffel et al. [42]^b^	Switzerland	High SDI	2023	3–6 months, 12.7% 6 months–1 years, 34.1% 1–2 years, 25.4% > 2 years, 27.8%	Oesophageal cancer	Stage I, 18.2% Stage II, 22.2% Stage III, 42.1% Stage IV, 17.5%	70.2	Not reported	Not reported	Not reported
17	Tsimicalis et al. [43]^d^	Canada	High SDI	2006–2008	Not reported	Acute lymphoblastic leukaemia 47 (46) Acute myeloid leukaemia 7 (7) Lymphomas 11% Central nervous system 10% Renal tumours 6% Malignant bone tumours 7% Other 12%	Not reported	7.85	Mother 37.38 Father 40.01	Not reported	Not reported
18	Van Houtven et al. [44]^b^	USA	High SDI	2005	Not reported	Lung cancer and colorectal cancer	Stage I 26.9% Stage II 19.8% Stage III 31.1% Stage IV 17.8%	> 65 years: 51.9%	> 65 years: 42.1%	Spouse 63.8% Other relative 32.4% Friend 3.8%	Not reported
19	Van Houtven et al. [45]^b^	USA	High SDI	Not reported	Not reported	Not reported	Advanced stage cancer	61.27	56	Not reported	Male 26% Female 74%
20	Wood et al. [46]^b^	France, Germany, Italy	High SDI	2015–2016	Not reported	Advanced non‐small cell lung cancer (NSCLC)	Stage III, 7% Stage IV, 93%	66.2	53.5	Partner/spouse 54.9% Parent 0.7% Friend/neighbour 3.1% Child 31.9% Sibling 2.6% Other family members 2.8% Other 4.0%	Female, 72.6%
21	Yabroff et al. [47]^b^	USA	High SDI	2000–2003	25.1 months	Breast Colorectal Prostage Lung Ovarian NLH Kidney Other (bladder, skin, uterine)	Not reported	Not reported	< 24, 10 1.5% 25–34, 40 5.8% 35–44, 85 12.4% 45–54, 223 32.4% 55–64,180 26.2% 65,131 19.0%	Spouse or partner 451, 65.6% Child (child‐in‐law) 111, 16.1% Parent 29, 4.2% Sibling 57, 8.3% Friend 26, 3.8% Other 14, 2.0%	Male 238 34.6% Female 450 65.4%
22	Yang et al. [48]^b^	China	High middle SDI	2020–2021	16.36 months	Non‐small cell lung cancer	Stage III Stage IV	≤ 60, 36.5% 60+, 63.5%	≤ 60, 75.3% ≥ 60, 24.7%	Spouse 296 (48.4) Daughter/Son 251 (41.1) Mother/Father 31 (5.1) Others 33 (5.4)	Not reported
23	Cicin et al. [49]^d^	Turkey	High middle SDI	Not reported	Not reported	Lung cancer	Not reported	Not reported	Not reported	Not reported	Not reported
24	Eze et al. [50]^b^	Nigeria	Low middle SDI	Not reported	8 months	Not reported	Not reported	Not reported	41.63	Child 26.7% Parent 13.3% Sibling 20% Spouse 17.1% Others 22.9%	Male 37.1% Female 62.9%
25	Hutchinson et al. [51]^d^	Australia, Canada UK USA Kazakhstan Malaysia, Colombia India Kenya Nigeria, Malawi	High High High High High‐middle High‐middle Middle Low middle Low middle Low middle Low	2023	Not reported	Ovarian Cancer	Not reported	Not reported	Not reported	Not reported	Not reported
26	Luengo‐Fernandez [52]^d^	27 countrues from European Union^a^	High and High‐middle	2009	Not reported	Breast cancer	Not reported	Not reported	Not reported	Not reported	Not reported

Abbreviations: SDI, Socio demographic index; UK, United Kingdom; USA, United States of America.

^a^ High SDI: Austria, Belgium, Cyprus, Czech Republic, Denmark, Estonia, Finland, France, Germany, Greece, Ireland, Italy, Latvia, Lithuania, Luxembourg, Netherlands, Poland, Slovenia, Spain, Sweden, UK High middle SDI: Bulgaria, Hungary, Italy, Malta, Portugal, Romania, Slovakia.

^b^ Cross‐sectional study.

^c^ Cohort study.

^d^ Cost of illness study.

Time since cancer diagnosis ranged from less than 6 months [29] to an average of 5.4 years [34]. Studies reported unpaid care hours and/or costs for various cancer types, including breast [39], lung [30, 46, 48, 49], colorectal [32, 33], head and neck [34], ovarian [51], prostate [37], oesophageal [42], as well as multiple carcinoma types [29, 31, 40, 41, 43, 44, 47]. Among these studies, one focused on children with cancer [43], while the mean age of adult patients ranged from 63 [29] to 76 years [28] in the other 14 studies [28, 29, 30, 31, 32, 34, 37, 39, 41, 42, 43, 44, 46, 48] (the remaining studies did not report the mean age of patients).

A total of 15 (58%) studies [28, 29, 30, 32, 34, 36, 37, 41, 43, 44, 45, 46, 47, 48, 50] reported carers' age, with mean values ranging from 37.8 to 59.15 years. Fourteen (48%) studies provided carers' gender [28, 29, 30, 32, 34, 36, 37, 40, 41, 44, 45, 46, 47, 50], with females comprising the majority in all studies (mean 78% in high and 62.9% from one study in low‐middle SDI) (Table S2). Spouses were the most commonly reported carers (percentage ranging from 25% [29] to 72% [32]).

Nine (35%) studies were conducted among advanced or palliative care patients [28, 29, 30, 31, 40, 41, 45, 46, 48].

### 3.1 Risk of Bias

The cross‐sectional studies scored generally well in defining the criteria for inclusion in the sample, describing the study subjects and settings, measuring outcomes in a valid and reliable way, using objective standard criteria for measurement of the condition and using appropriate statistical tests [27, 29, 32, 33, 34, 36, 37, 38, 39, 40, 41, 42, 44, 45, 46, 47, 48, 50]. As these were observational studies with no interventions, exposure measurement was not applicable across any study [27, 29, 32, 33, 34, 36, 37, 38, 39, 40, 41, 42, 44, 45, 46, 47, 48, 50]. Moreover, none of the studies addressed confounding factors, and consequently, strategies for managing confounding were not applicable [27, 29, 32, 33, 34, 36, 37, 38, 39, 40, 41, 42, 44, 45, 46, 47, 48, 50].

Similarly, the cohort studies demonstrated strong performance in outcomes measurement, follow‐up time, completion of follow‐up and using statistical tests [28, 30, 31]. However, the similarity of groups of patients was not applicable due to the absence of comparison groups, exposure measurement was not relevant due to the lack of intervention, and no confounding factors were identified in any of the studies [28, 30, 31].

Among the cost‐of‐illness studies [35, 43, 49, 51, 52], study characteristics, methodology and cost analysis were generally well defined. In particular, studies [43, 49] clearly defined the epidemiological approach (prevalence or incidence‐based). However, limitations were noted and included unclear data collection approach (prospective or retrospective) [49, 51, 52], unclear or not performed sensitivity analysis [49, 51], and discounting was not applied as the analysis was limited to 1 year [35, 43, 49]. Nonetheless, results were reported clearly across studies (Table S3).

### 3.2 Unpaid Caregiving Duration and Caregiving Hours

Table 2 reports unpaid cancer caregiving hours per week. Among the eight [29, 31, 36, 38, 44, 47, 48, 50] studies that reported the caring period, the duration ranged from less than three months [48] to 3.07 years [36] with some caregivers in one study reporting more than 4 years of caregiving [38]. In studies that reported daily unpaid care hours [27, 29, 35, 41, 45, 47, 48, 50], daily hours ranged from 1 h [35] to 14 h [50]. Most studies reported the unpaid hours on a weekly basis [31, 34, 36, 37, 39, 42, 44, 46, 51], while the remaining studies reported over two weeks [28] or a three‐month period [40, 43].

**TABLE 2:** Results of informal caregiving hours.

Sr No	Study details	Country	Region	Informal caregiving duration	Caregiving hours as reported in the study	Caregiving hours per week (calculated where not reported in weeks)^a^	Method used to collect caregiving time
1	Bayen et al. [27]	France	High SDI	Not reported	11.7 h per day	81.90 h per week^1^	Not reported
2	Cai et al. [28]	Canada	High SDI	Not reported	Days till death hours per 2 weeks 0–30^a^: 141.31 h 31–60: 127.11 h 61–90: 115.14 h 91–180: 103.13 h 181–365: 89.52 h > 365: 84.49 h	54.60 h per week^2,3^	Interviews
3	Chua et al. [29]	Singapore	High SDI	< 6 months (31.3%) 6 months–1 year (6.3%) 1–2 years (12.5%) 2 years (50%)	Hours per day < 4 h: 0% 4–8 h: 7.7% 8–12 h: 15.4% 12 h: 76.9%	84 h per week^1,4^	Survey
4	Haltia et al. [31]	Finland	High SDI	Breast: 59 days Colorectal: 181 days Prostate: 239 days All: 179 days	11 h per week	11 h per week	Questionnaires
5	Hanly et al. [33]	Ireland	High SDI	Not reported	Diagnosis, initial treatment phase: 42.5 h per week out of which Hospital‐related activities 13.9 and Domestic‐related activities 28.6 Ongoing care phase: 16.9 h per week	29.7 h per week^5^	Questionnaires
6	Hanly et al. [34]	Ireland	High SDI	Not reported	17.8 per week	17.8 h per week	Questionnaire and survey
7	Hao et al. [35]	Sweden	High SDI	Not reported	Severely limited Overall: 1–1.1 h per day < 65 year = 0.5 h–0.6 h per Terminally ill phase Overall: 4 h per week Caregiver < 65 year: 0.5–0.6 h per week	17.5 h per week^1,5^	Survey of Health, Ageing and Retirement in Europe (SHARE)
8	Hendricks et al. [36]	USA	High SDI	3.07 years	22.43 per week	22.43 per week	Survey
9	Li et al. [37]	USA	High SDI	Not reported	1.3 h per week	1.30 h per week	Self‐administered survey
10	Roine et al. [39]	Finland	High SDI	Not reported	Primary treatments 4.5 h per week Rehabilitation 0.9 h per week Remission 0.3 h per week Metastatic disease 6.1 h per week	2.95 h per week^5^	Questionnaires
11	Rowland et al. [40]	UK	High SDI	Not reported	95 h per 3 months	7.92 h per week^6^	Mailed surveys
12	Spatuzzi et al. [41]	Italy	High SDI	Not reported	Caregiver < 65 years 24 h per day 35.1% 3 h per day 2.1% 4–6 h per day 22.3% 7–12 h per day 37.2% Night 3.2% Caregiver > 65 years 24 h per day 40% 3 h per day 8.8% 4–6 h per day 11.3% 7–12 h per day 37.5% Night 2.5%	168 h per week^4^	Not reported
13	Stoffel et al. [42]	Switzerland	High SDI	Not reported	All caregivers Overall 15.8 h per week Active stage 18.2 h per week Caregiver < retirement age Overall 6.9 h per week Active stage 9.3 h per week	15.8 h per week^7^	Questionnaires
14	Tsimicalis et al. [43]	Canada	High SDI	Not reported	1365 h of care in 3 months	113.8 h per week^8^	Not reported
15	Van Houtven et al. [44]	USA	High SDI	Initial phase: 29.9 weeks Continuing phase: 71.5 weeks Terminal phase: 37.0 weeks Any phase: 51.5 weeks	Initial phase: 15.3 h per week Continuing phase: 15 h per week Terminal phase: 24.5 h per week Any phase: 16 h per week	16.00 h per week^7^	Survey
16	Van Houtven et al. [45]	USA	High SDI	Not reported	10.21 per day	71.5 per week	Questionnaire
17	Wood et al. [46]	France	High SDI	Not reported	24.2 h per week	24.20 h per week	Caregiver questionnaire
17	Wood et al. [46]	Germany	High SDI	Not reported	10.8 h per week	10.80 h per week	Caregiver questionnaire
17	Wood et al. [46]	Italy	High SDI	Not reported	40.1 h per week	40.10 h per week	Caregiver questionnaire
18	Yabroff et al. [47]	USA	High SDI	All cancers average 13.7 months Breast 13.6 months Colorectal 13.5 months Prostate 12.5 months Lung 16.1 months Ovarian 16.7 months NHL 15.2 months Kidney 11.4 months Other 12.1 months Based on cancer stage Localised: 12.2 months Regional: 14.5 months Distant: 17.9 months	All cancers average 8.3 h per day Breast: 6.4 h per day Colorectal: 8.2 h per day Prostate: 9.1 h per day Lung: 10.8 h per day Ovarian: 10.3 h per day NHL: 10.7 h per day Kidney: 9.8 h per day Other: 6.8 h per day Based on cancer stage Localised: 7.8 h per day Regional: 8.3 h per day Distant: 9.8 h per day	58.10 h per week^1,7^	American Cancer Society's Quality of Life Survey
19	Hutchinson et al. [51]	Australia	High SDI	Not reported	Pretreatment 7.0 h per week Active treatment 38.9 h per week Maintenance/monitoring 16.9 h per week Palliative care 7.6 h per week	17.60 h per week^5^	Meta‐analysis^b^
19	Hutchinson et al. [51]	Canada	High SDI				
19	Hutchinson et al. [51]	UK	High SDI				
19	Hutchinson et al. [51]	USA	High SDI				
					Average High SDI	38.3 h per week	
20	Yang et al. [48]	China	High middle SDI	< 3 months: 187 3–6 months: 139 7–12 months: 88 > 12 months: 197	< 3 h per day: 31.8% 3–6 h per day: 23.7% 7–9 h per day:: 9.2% > 9 h per day: 35.4%	63.00 h per week^4^	Face‐to‐face interviews using questionnaires
21	Hutchinson et al. [51]	Kazakhastan	High middle SDI	Not reported	Pretreatment 7.0 h per week Active treatment 38.9 h per week Maintenance/monitoring 16.9 h per week Palliative care 7.6 h per week	17.60 h per week^5^	Meta‐analysis^b^
21	Hutchinson et al. [51]	Malaysia	High middle SDI				
					Average High middle SDI	32.7 h per week	
21	Hutchinson et al. [51]	Colombia	Middle SDI		Pretreatment 7.0 h per week Active treatment 38.9 h per week Maintenance/monitoring 16.9 h per week Palliative care 7.6 h per week	17.60 h per week^5^	Meta‐analysis^b^
					Average Middle SDI	17.6 h per week	
21	Hutchinson et al. [51]	India	Low middle SDI	Not reported	Pretreatment 7.0 h per week Active treatment 38.9 h per week Maintenance/monitoring 16.9 h per week Palliative care 7.6 h per week	17.60 h per week^5^	Meta‐analysis^b^
21	Hutchinson et al. [51]	Kenya	Low middle SDI				
21	Hutchinson et al. [51]	Nigeria	Low middle SDI				
22	Eze et al. [50]	Nigeria	Low middle SDI	6 months	14.92 per day	104.44 per week	
					Average Low middle SDI	38.3 h per week	
21	Hutchinson et al. [51]	Malawi	Low SDI	Not reported	Pretreatment 7.0 h per week Active treatment 38.9 h per week Maintenance/monitoring 16.9 h per week Palliative care 7.6 h per week	17.60 h per week^5^	Meta‐analysis^b^
					Average Low SDI	17.6 h per week	

Abbreviations: SDI, Socio demographic index; UK, United Kingdom; USA, United States of America.

^a^ Weekly hours calculation method: 1. Daily hours were multiplied by 7 to calculate weekly hours 2. Median hours was used 3. Hours over 2 weeks was divided by 2 to calculate weekly hours 4. Data for highest proportion of respondents was considered and if there were two such categories, average was calculated 5. Average of all phases/patient types was calculated 6. Data over 3 months was divided by 12 to calculate weekly data 7. Data for overall was used 8. Data was divided by 12 for weekly hours.

^b^ Meta‐analysis of the hours per week of unpaid caregiving provided by treatment phase. It used the time‐use estimates collected from the 21 studies, 13 unique cancers and 11 unique countries (9 high income, two upper middle income).

Studies that reported unpaid care hours were conducted mainly in high SDI countries, except for two that were conducted in high‐middle SDI [48, 49], one in low‐middle SDI [50] and two that were conducted across multiple countries with differing SDI levels [51, 52]. The weekly mean unpaid care hours in the high SDI countries was 38.3 (range 1.3 [37] to 113.8 [43]) across 23 studies (which included 21 countries), 32.7 in high‐middle, 39.3 in low‐middle [50] and 17.6 in the middle and low SDI countries [51] (Table S2).

Unpaid care hours also varied with cancer type and stage. The study reporting the lowest unpaid care hours was conducted in men with localised prostate cancer [37], whereas the highest hours were reported in advanced‐stage cancer [29]. Additionally, Yabroff et al. reported that people with lung cancer required the highest unpaid care hours, while those with breast cancer required the least [47]. Moreover, eight of the included studies focused on patients who received palliative care, and among these, the unpaid care hours ranged from 24.5 [46] to 84 per week (hours reported by most respondents) [29].

Questionnaires or surveys were the most common method used to elicit unpaid care time [29, 31, 32, 33, 34, 36, 37, 38, 39, 40, 42, 44, 45, 46, 48, 50]. Among these, two used data from longitudinal surveys: the American Cancer Society's Quality of Life Survey [47] and the Survey of Health, Ageing and Retirement in Europe (SHARE) [35, 52].

### 3.3 Exploratory Meta‐Analysis of Unpaid Care Hours

A total of eight studies [33, 34, 37, 40, 43, 45, 47, 50] reported the mean, sample size, SD/95% CI of unpaid care hours and were therefore included in the exploratory meta‐analysis. The pooled unpaid care hours per week based on the random effects model were 48.35 (95% CI: 17.81–78.89) with high heterogeneity (*I* ^2^ = 100%) (Tables S4 and S5). Due to the limited number of studies, a subgroup analysis by SDI could not be conducted. The high heterogeneity was most likely attributable to substantial variations in study populations, different cancer types and stages being cared for, study methods, and the timeframe used for measuring the unpaid care hours.

### 3.4 Unpaid Care Costs

The cancer unpaid care costs are reported in Table 3. Nine studies used the opportunity cost [32, 35, 37, 43, 44, 47, 48, 51, 52] and five used replacement cost approaches [30, 31, 38, 39, 42], with two using both methods [33, 34] to calculate unpaid care costs. The unit cost for unpaid care ranged from US$27 [31, 39] to US$55 per hour [30] across all studies. The average unit cost was US$19 and US$23 (using opportunity and replacement cost approach, respectively) in high SDI countries, US$6 in high middle SDI, US$1.5 in middle SDI, US$0.6 in low middle SDI countries and US$2.1 in low SDI (only one country represented for middle and low SDI classifications respectively) (Table S2).

**TABLE 3:** Results of informal care costs.

Sr. no.	Study details	Country	Region	Method used to calculate caregiving cost	Informal caregiving cost per hour (cost in USD 2024)	Mean informal care cost per capita Country level costs (2024 US$)
1	Bayen et al. [27]	France	High SDI	Not reported	Not reported	PGA Є677 ($946) per month/Є8124 (11347) annually OCA Є1683 ($2351) per month/Є20,196 (28208) annually
2	Gridelli et al. [30]	Italy	High SDI	PGA	Nurse €34.8 ($55) Physiotherapist €20.66 ($33) Housekeeper €4.33 ($7)	Main caregiver (over 3 months) 2nd line chemotherapy €2368 ($3776) Supportive care €2805 ($4472)
3	Haltia et al. [31]	Finland	High SDI	PGA: Labour market value	€18.89 ($27.07)	During the days of palliative care Breast: €3130 ($4486) Colorectal: €7604 ($10,897) Prostate: €25,205 ($36,122) All: €5951 ($8529)
4	Hanly et al. [32]	Ireland	High SDI	OCA: Average wage per hour	OCM: €21.21 ($31.03)	Mean weekly indirect cost Diagnosis and treatment phase: €608.7 ($890.6) Ongoing care phase: €352.7 ($516)
5	Hanly et al. [33]	Ireland	High SDI	OCA, PGA variants and mixed (OCA & PGA) PGM: Health‐care assistant and home‐help Mixed: Various wage rates	OCA: €21.21 ($29.6) (base case) PGA: €16.09 ($22.5) *Only the first reported unit cost was included, this study used various other unit costs	Weekly time costs (base case) Diagnosis and initial treatment phase: Hospital‐related activities 295 ($412) Domestic‐related activities 630 ($880) Ongoing care phase 359 ($501)
6	Hanly et al. [34]	Ireland	High SDI	OCA: Individual's market wage PGA (GRCA) and SRCA: Various wage rates	OCA: €22.30 ($30.5) PGA: €14.63 ($20) 7*Only the first reported unit cost was i8ncluded, this study used various other unit costs	Per year OCA:€20,613 ($28,200) GRCA:€13,196 ($18,053) SRCA:€14,196 ($19,421)
7	Hao et al. [35]	Sweden	High SDI	OCA	€28 ($37)	*Note: Did not report mean patient cost but reported the total cost of the country Stockholm region €18,120,816 ($24,230,333) Sweden €89,142,341 ($119,197,094)
8	Li et al. [37]	USA	High SDI	OCA: Median income of women over 55 years and older	Not reported	Per year $6063 ($8973)
9	Palandri et al. [38]	Italy	High SDI	PGA	€4.4/$5.21 ($7.1) or €7.83/$9.26 ($12.6)	Annual: 20326 €/$27,550 ($37,468)
10	Roine et al. [39]	Finland	High SDI	PGA: Average wage rates	€18.89 ($27)	Per 6 months Primary treatments €2191 ($3140) Rehabilitation €453 ($649) Remission €166 ($238) Metastatic disease €2985 ($4278)
11	Stoffel et al. [42]	Switzerland	High SDI	PGA	Not reported	Per 4 weeks Overall: CHF360 ($348) Active stage: CHF 404 (391)
12	Tsimicalis et al. [43]	Canada	High SDI	OCA	Paid sick or vacation hours and forgone, non‐reimbursable, market labour hours: CAD11.14 ($22) to CAD24.21 ($47) per h for women and CAD12.46 ($24) to CAD24.43 ($48) for men Time spent away from other activities: national average hourly wage of a child care giver $CAD14.61 ($29)	3 months Unpaid activities Mother CAD11373 ($22,220) Father CAD4985 ($9739) Loss of work‐non reimbursed Mother CAD2295 ($4484) Father CAD1876 ($3665)
13	Van Houtven et al. [44]	USA	High SDI	OCA	Caregiver of initial phase patient $13.3 ($21.8) Caregiver of initial phase patient $18.8 ($30.8) Caregiver of terminal phase patient $15.7 ($25.7) Caregiver of patient in any disease phase $15.7 ($25.7)	Caregiver of initial phase patient $5939 ($9740) Caregiver of continuing phase patient $17,942 ($29,425) Caregiver of terminal phase patient $12,981 ($ 21,289) Caregiver of patient in any disease phase $12,618 ($20,694)
14	Yabroff et al. [47]	USA	High SDI	OCA: National median wage	National median wage $16.78 ($27)	Over 2 years All $47,710 ($75,382) Lung $72,702 ($114,869) Colorectal $45,699 ($72,204) Prostate 44,885 ($70,918) Ovarian $66,210 ($104,612) NHL $59,613 ($94,189) Kidney 53,541 ($84,595) Breast $38,334 ($60,568) Stage wise Localised disease $40,973 ($$64,737) Regional $51,091 ($80,724) Distant disease $71,278 ($112,619)
15	Hutchinson et al. [51]	Australia	High SDI	OCA: National minimum wages	$14 ($15.5)	*Note: Did not report mean patient cost but reported the total cost for the country $33.0 ($36.6) million
15	Hutchinson et al. [51]	Canada	High SDI	OCA: National minimum wages	$10.96 ($12.2)	*Note: Did not report mean patient cost but reported the total cost for the country $69.3 ($76.9) million
15	Hutchinson et al. [51]	UK	High SDI	OCA: National minimum wages	$11.20 ($12.4)	*Note: Did not report mean patient cost but reported the total cost for the country $95.7 ($106.2) million
15	Hutchinson et al. [51]	USA	High SDI	OCA: National minimum wages	$7.25 ($8)	*Note: Did not report mean patient cost but reported the total cost for the country $228.0 ($253.1) million
16	Luengo‐Fernandez [52]	Austria	High SDI	OCA	Employed; Unemployed: €16 ($23); €10 ($14)	*Note: Did not report mean patient cost but reported the total cost for the country €550 ($792) million
16	Luengo‐Fernandez [52]	Belgium	High SDI	OCA	Employed; Unemployed: €21 ($30); €9 ($13)	*Note: Did not report mean patient cost but reported the total country level cost €553 ($796) million
16	Luengo‐Fernandez [52]	Cyprus	High SDI	OCA	Employed; Unemployed: €13 ($19); €6 ($9)	*Note: Did not report mean patient cost but reported the total country level cost €15 ($22) million
16	Luengo‐Fernandez [52]	Czech Republic	High SDI	OCA	Employed; Unemployed: €6 ($9); €2 ($3)	*Note: Did not report mean patient cost but reported the total country level cost €122 ($176) million
16	Luengo‐Fernandez [52]	Denmark	High SDI	OCA	Employed; Unemployed: €29 ($42); €11 ($16)	*Note: Did not report mean patient cost but reported the total country level cost €277 ($399) million
16	Luengo‐Fernandez [52]	Estonia	High SDI	OCA	Employed; Unemployed: €5 ($7); €2 ($3)	*Note: Did not report mean patient cost but reported the total country level cost €17 ($24) million
16	Luengo‐Fernandez [52]	Finland	High SDI	OCA	Employed; Unemployed: €19 ($27); €11 ($16)	*Note: Did not report mean patient cost but reported the total country level cost €166 ($239) million
16	Luengo‐Fernandez [52]	France	High SDI	OCA	Employed; Unemployed: €16 ($23); € 9 ($13)	*Note: Did not report mean patient cost but reported the total country level cost €2543 ($3661) million
16	Luengo‐Fernandez [52]	Germany	High SDI	OCA	Employed; Unemployed: €23 ($33); €10 ($14)	*Note: Did not report mean patient cost but reported the total country level cost €6414 ($9233) million
16	Luengo‐Fernandez [52]	Greece	High SDI	OCA	Employed; Unemployed: €15 ($22); €6 ($9)	*Note: Did not report mean patient cost but reported the total country level cost €348 ($501) million
16	Luengo‐Fernandez [52]	Ireland	High SDI	OCA	Employed; Unemployed: €22 ($32); €10 ($14)	*Note: Did not report mean patient cost but reported the total country level cost €162 ($233) million
16	Luengo‐Fernandez [52]	Latvia	High SDI	OCA	Employed; Unemployed: €4 ($6); €2 ($3)	*Note: Did not report mean patient cost but reported the total country level cost €23 ($33) million
16	Luengo‐Fernandez [52]	Lithuania	High SDI	OCA	Employed; Unemployed: €4 ($6); €2 ($3)	*Note: Did not report mean patient cost but reported the total country level cost €29 ($42) million
16	Luengo‐Fernandez [52]	Luxembourg	High SDI	OCA	Employed; Unemployed: €29 ($42); €11 ($16)	*Note: Did not report mean patient cost but reported the total country level cost €26 ($37) million
16	Luengo‐Fernandez [52]	the Netherlands	High SDI	OCA	Employed; Unemployed: €22 ($32); €9 ($13)	*Note: Did not report mean patient cost but reported the total country level cost €983 ($1415) million
16	Luengo‐Fernandez [52]	Poland	High SDI	OCA	Employed; Unemployed: €5 ($7); €2 ($3)	*Note: Did not report mean patient cost but reported the total country level cost €550 ($792) million
16	Luengo‐Fernandez [52]	Slovenia	High SDI	OCA	Employed; Unemployed: €9 ($13); €4 ($6)	*Note: Did not report mean patient cost but reported the total country level cost €42 ($60) million
16	Luengo‐Fernandez [52]	Spain	High SDI	OCA	Employed; Unemployed: €13 ($19); €5 ($7)	*Note: Did not report mean patient cost but reported the total country level cost €1581 ($2276) million
16	Luengo‐Fernandez [52]	Sweden	High SDI	OCA	Employed; Unemployed: €18 ($26); €12 ($17)	*Note: Did not report mean patient cost but reported the total country level cost €397 ($571) million
16	Luengo‐Fernandez [52]	UK	High SDI	OCA	Employed; Unemployed: €16 ($23); €7 ($10)	*Note: Did not report mean patient cost but reported the total country level cost €2334 ($3360) million
17	Yang et al. [48]	China	High SDI	OCA: Daily income of caregivers	Not reported	Per capita cost since diagnosis (16.36 months) $1151 ($1393)
18	Cicin et al. [49]	Turkey	High middle SDI	Not reported	Minimum income $501 ($631)/month, retirement pension $312 ($393)/month and disability retirement pension $204 ($257)/month	€29,906,627 ($39,056,884) for 47,500 persons
15	Hutchinson et al. [51]	Kazakhstan	High middle SDI	Not reported	$0.75 ($0.8)	*Note: Did not report mean patient cost but reported the total country level cost $1.2 ($1.3) million
15	Hutchinson et al. [51]	Malaysia	High middle SDI	Not reported	$1.97 ($2.2)	*Note: Did not report mean patient cost but reported the total country level cost $6.3 ($7) million
16	Luengo‐Fernandez [52]	Bulgaria	High middle SDI	OCA	Employed; Unemployed: €2 ($3); €1 ($1)	*Note: Did not report mean patient cost but reported the total country level cost €31 ($45) million
16	Luengo‐Fernandez [52]	Hungary	High middle SDI	OCA	Employed; Unemployed: €5 ($7); €2 ($3)	*Note: Did not report mean patient cost but reported the total country level cost €122 ($176) million
16	Luengo‐Fernandez [52]	Italy	High middle SDI	OCA	Employed; Unemployed: €15 ($22); €7 ($10)	*Note: Did not report mean patient cost but reported the total country level cost €5491 ($7905) million
16	Luengo‐Fernandez [52]	Malta	High middle SDI	OCA	Employed; Unemployed: €9 ($13); €4 ($6)	*Note: Did not report mean patient cost but reported the total country level cost €9 ($13) million
16	Luengo‐Fernandez [52]	Portugal	High middle SDI	OCA	Employed; Unemployed: €10 ($14); €3 ($4)	*Note: Did not report mean patient cost but reported the total country level cost €268 ($386) million
16	Luengo‐Fernandez [52]	Romania	High middle SDI	OCA	Employed; Unemployed: €3 ($4); €1 ($1)	*Note: Did not report mean patient cost but reported the total country level cost €112 ($161) million
16	Luengo‐Fernandez [52]	Slovakia	High middle SDI	OCA	Employed; Unemployed: €5 ($7); €2 ($3)	*Note: Did not report mean patient cost but reported the total country level cost €53 ($76) million
16	Hutchinson et al. [51]	Colombia	Middle SDI	Not reported	$1.36 ($1.5)	*Note: Did not report mean patient cost but reported the total country level cost $4.4 ($4.9) million
16	Hutchinson et al. [51]	India	Low Middle SDI	Not reported	$0.34 ($0.4)	*Note: Did not report mean patient cost but reported the total country level cost $28 8.0 ($32) million
16	Hutchinson et al. [51]	Kenya	Low Middle SDI	Not reported	$0.79 ($0.9)	*Note: Did not report mean patient cost but reported the total country level cost $1.8 ($2.8) million
16	Hutchinson et al. [51]	Nigeria	Low Middle SDI	Not reported	$0.4 ($0.4)	*Note: Did not report mean patient cost but reported the total country level cost $2.5 ($2.8) million
16	Hutchinson et al. [51]	Malawi	Low SDI	Not reported	$1.85 ($2.1)	*Note: Did not report mean patient cost but reported the total country level cost $0.8 ($0.9) million

Abbreviations: CAD, Canadian dollar; CHF, Swiss franc; GRCA, Generalist replacement cost approach; OCA, Opportunity cost approach; PGA, Proxy good approach (or replacement cost approach); SDI, Socio demographic index; SRCA, Specialist replacement cost approach; UK, United Kingdom; USA, United States of America.

Reported timeframes for the unpaid care cost estimation varied from one week [32, 33] to 1 year [34, 37, 44]. The average unpaid care cost was US$2249 per month (range US$346 [39] to US$5626 [32]) for high SDI countries. The calculated average monthly unpaid care cost was US$196, US$26, US$24 and US$37 in high‐middle, middle, low‐middle and low SDI, respectively (Table 4). Studies that included multiple SDI countries reported aggregate national [51] or regional (e.g., European Union) [52] costs rather than per‐patient averages, inhibiting cross‐study averaging.

**TABLE 4:** Mean by SDI: Informal care hours, unit cost and informal care cost.

	Informal care hours per week	Informal care unit cost (2024$USD)	Informal care cost per month (2024US$)
High SDI	38.3	$23^c^ and $19^d^	$2249
High middle SDI	32.7	$6^d^	$196^b^
Middle SDI	17.6^a^	$1.5^a^, ^d^	$26^b^
Low middle SDI	39.3	$0.6	$23.6^b^
Low SDI	17.6^a^	$2.1^a^	$37^b^

Abbreviations: NA, Not available; SDI, Socio demographic index.

^a^ One study which included one country data.

^b^ Calculated by multiplying informal care hours per week and informal care unit cost (2024$) from this table.

^c^ PGA: Proxy good approach (or replacement cost approach).

^d^ Opportunity cost approach.

A summary of unpaid care hours and costs is provided in Table 4.

## 4 Discussion

To our knowledge, this is the first study to explore the unpaid cancer care time and cost burden by global income levels based on the SDI classification of countries. Our review highlights a significant evidence gap in the impact of cancer caregiving roles in low, low‐middle and middle SDI countries. On average unpaid cancer carers in low‐middle SDI countries spent 39.3 h per week, followed by 38.3 in high SDI countries, 32.7 for high‐middle SDI, 17.6 for middle and low SDI countries. However, this difference may reflect data limitations rather than true variation. The estimates for middle and low SDI regions were based on a single study that assumed uniform caregiving hours across regions but applied different unit costs [51] and that for low‐middle was based on two studies [50, 51]. This highlights the severe lack of data from lower‐income countries.

Notably, unpaid cancer care hours appeared to be largely in line with other chronic conditions. Previous studies reported the pooled unpaid care hours per week of 25.8 in stroke [53] and an average of 44.1 in dementia [54]. These earlier studies were predominantly conducted in high SDI countries, except for one study each from India, Malaysia, China and Thailand in stroke (out of 31 studies) [53] and Turkey in dementia (out of 111 studies) [54] highlighting the lack of unpaid care data from lower SDI countries in other disease areas as well. The wide range of unpaid care hours observed in our review is reflected in the high heterogeneity in the exploratory meta‐analysis. These were likely to be attributed to variation in cancer types, stages of cancer and settings.

The opportunity cost was the most commonly used method for estimating unpaid care costs, followed by replacement cost and hybrid approaches. Some studies applied refined methodologies, such as the generalised replacement cost approach (GRCA) or the specialist replacement cost approach (SRCA) [33, 34]. These methods account for sociodemographic and economic characteristics of the carer population, which are especially relevant given that unpaid carers are predominantly older women with lower labour force participation rates [34]. These same patterns are common in cancer carer populations [34, 55] and so were reported in our review using GRCA or differentiated wages based on SRCA. Given the typically reduced workforce engagement in this group, applying a zero or minimum wage to unemployed carers risks underestimating the true societal cost of care [34].

The wide variation in unpaid care costs is consistent with differences in reported caregiving hours. Among high SDI countries, the mean monthly unpaid care cost was US$2249‐comparable to a previous scoping review that reported CA$3843 (equivalent to US$2802) [13]. In our review, hourly unit cost ranged from $27 [31, 39] to $55 [30], with the mean value of $19 for the opportunity cost approach and $23 for the replacement cost approach. These are slightly higher than corresponding estimates from studies on dementia ($15 and $18.17) and stroke ($23.47 and $17.47), and may be attributed to averaging multiple unit costs within studies [30, 33, 38, 43, 44, 51, 52].

While high SDI countries still report higher cancer incidence due to longer life expectancy [56] and better diagnostic systems [57], cancer burden is disproportionately higher in lower SDI countries due to potentially delayed diagnoses, limited access to advanced treatment and under‐resourced healthcare systems [58, 59]. Lower unpaid care costs in lower SDI countries may be driven by multiple factors, including lower wage rates, lack of quality data systems [60] and shorter caregiving durations due to poorer cancer survival (as compared to high SDI countries) [61]. Additionally, data collection in these regions is often limited by resource constraints, inconsistent methodologies and inadequate long‐term follow‐up [62]. Access to early detection programs is also poor, contributing to delayed presentation. For example, the median patient delay in cancer diagnosis is 6.5 months in lower‐middle‐income countries, compared to 1 month in upper‐middle‐income countries [57, 63, 64]. Although cancer incidence is generally lower in less developed countries, mortality rates are disproportionately higher, sometimes 8%–15% higher than in high‐income countries [64]. As the demographic of lower SDI countries shifts to an ageing population, the burden of non‐communicable diseases, including cancer, is expected to rise following a similar trajectory observed historically in high SDI countries [65]. Adding to this burden is the cancer incidence associated with preventable risk factors such as obesity, more typically seen in high SDI countries, now growing in low‐middle SDI countries with rapidly expanding economies [65]. The caregiving burden in terms of time or cost in these countries needs to be explored further and monitored over time.

We utilised SDI as a metric to categorise the countries, which incorporates total fertility rate under the age of 25, mean education for those ages 15 and older, and lag distributed income per capita [22]. Due to the inclusion of these factors, it has long been used as a composite socio‐demographic parameter that defines the social and economic contexts relevant to health [66]. However, access to cancer screening, diagnosis and treatment may vary within a country irrespective of its SDI categorisation. High SDI countries continue to face disparities in access to care among populations experiencing socioeconomic disadvantage [67]. Barriers to cancer treatment operate across multiple levels, including inadequate or absent insurance coverage, and geographical distribution of services at the macro level; limited social care support, poor quality of communication between patient and cancer care providers, and lack of care continuity at the meso level; and poverty, racism and unstable housing at the micro level [67]. These findings underscore the importance of identifying at‐risk patients in all geographies to improve access to treatment and cancer outcomes [67].

Caregiving situations vary substantially among countries and are shaped by health and social care policies, cultural values and societal norms [68, 69]. In several high‐income countries, including England, Canada and parts of the United States, formal mechanisms exist to financially compensate or reimburse family caregivers through paid and unpaid carer leave or paid caregiving schemes [70]. These policies recognize the economic value of unpaid care and aim to mitigate the financial burden borne by family caregivers. In Australia, Germany and the UK, National carer strategies outline government actions to support unpaid caregivers, including formal recognition of caregiving roles, employment protections (such as flexible work arrangements and leave entitlements), access to services and income support [70]. Canada is also progressing towards a National Caregiving Strategy that explicitly considers financial and social supports for family caregivers [71] Evidence suggests that the availability of formal care services may partially substitute for informal care [72], and caregiver support policies, such as allowances, tax credits, paid and unpaid leave, nursing fee and respite care, can reduce reliance on institutional care [73, 74]. In contrast, many Asian countries lack comprehensive policy frameworks for long‐term care services [75] and family caregiving remains the dominant model due to limited formal support, strong cultural expectations and societal norms regarding filial responsibility [69]. Finally, the country payer systems differ, where on one hand, there are countries with a hybrid, multiple‐payer system like the United States [76] and ‘single payer’ systems in the UK, Canada, Norway or Sweden, where national, regional, local government, or a combination of these entities is responsible for health care payment [77].

The demand for caregiving across all diseases is expected to increase in the coming decades. In the United States, the number of unpaid caregivers increased by 45% between 2015 and 2025, with almost one‐quarter of all American adults currently acting as family caregivers [78]. In Australia, the number of unpaid caregivers increased by 5.5% between 2018 and 2020 [79], while the total hours of unpaid care rose from 1.9 billion in 2015 [80] to 2.2 billion in 2020 [79]. In England and Wales, the proportion of individuals providing 20–49 h of care per week increased from 1.5% to 1.9%, and those providing 50 or more hours increased slightly from 2.7% to 2.8% between 2011 and 2021 [81]. Projections indicate that informal care hours and the number of care recipients will continue to rise. By 2039, England is expected to require 36% more informal care hours than in 2019 [82]. Similarly, the demand for unpaid carers in Australia is expected to increase by 23% between 2020 and 2030, from 1.25 million to 1.54 million [79].

This review provides a comprehensive synthesis of the global time and cost burden of unpaid caregiving. It is the first analysis to perform an exploratory meta‐analysis of unpaid care hours in this context. Despite inevitable heterogeneity in the included studies, our inclusive approach, covering all cancer types and stages, was designed to capture the full spectrum of caregiving time or cost burden. However, several limitations to our meta‐analysis are noted. Productivity losses among carers were excluded, as our focus was specifically on unpaid care provision. Secondly, there were a limited number of studies in lower SDI countries, which may result in underrepresentation of caregiving time and cost in these settings. Moreover, one study [51] used the same unpaid care hour data for all countries regardless of the SDI, which was not based on primary data gathered from the unpaid carers of that country, raising concerns about the accuracy and contextual validity of the estimates. We excluded studies reporting caregiving burden/health outcomes. Finally, most included studies were cross‐sectional in nature, limiting the ability to assess changes in caregiving time or cost burden over time or infer causality.

Certain limitations related to data searches and screening should be acknowledged, such as the exclusion of articles in languages other than English, which may have been published for studies conducted in lower‐income countries. Additionally, the use of the Research Screener poses certain limitations. Despite this platform being used by two independent reviewers and the extensive search through key databases, some articles may have been missed.

## 5 Conclusion

Unpaid cancer carers dedicate significant amounts of time to supporting individuals living with and beyond cancer, often providing complex and sustained care without financial compensation. The estimated economic value of this unpaid care is substantial and represents a critical, yet often overlooked, component of the cancer care continuum. We also highlight the significant gap in the evidence from lower‐income countries. To inform equitable and sustainable health system planning and productivity considerations, future research should further quantify the time and cost of unpaid cancer caregiving across diverse global regions, particularly in low‐ and middle‐income countries where evidence remains limited and resources for formal care are constrained. Recognising and integrating unpaid care into national cancer control strategies will be essential to ensure the resilience and inclusiveness of health systems globally.

## Author Contributions

Lan Gao: Supervision, methodology, validation, visualisation, writing – review and editing, project administration; Neha Das: Formal analysis, methodology, data curation, visualisation, writing – original draft; Shalika Bohingamu Mudiyanselage: Formal analysis, methodology, data curation, visualisation, writing – review and editing; Dr. Natalie Winter: Writing – review amd editing; Dr. Stephanie Cowdery: Writing – review and editing; Dr. Victoria White: Writing – review and editing; Dr. Patricia M. Livingston: Writing – review and editing.

## Funding

This work was supported in part by funding from the Victorian Government Department of Health, to the Centre of Excellence in Cancer Carer Research, Translation and Impact at Deakin University. Lan Gao received support of the Victorian Cancer Agency Mid‐Career Fellowship from the Victorian State Government. The funders played no role in the design, conduct, or interpretation of the findings of this study.

## Conflicts of Interest

Natalie Winter, Stephanie Cowdery and Victoria White received support for the present manuscript through payment made to the Cancer Strategy Unit, Victorian Department of Health. Lan Gao, Neha Das, Shalika Bohingamu Mudiyanselage and Patricia M. Livingston declare no conflicts of interest.

## Supporting information


**Table S1:** cam471657‐sup‐0001‐TablesS1‐S5.docx.

## Data Availability

The authors confirm that the data supporting the findings of this study are available within the article and Tables S1–S5.
