# Supplementary material for: Global Inequities in Unpaid Cancer Caregiving: A Systematic Review and Exploratory Meta‐Analysis of Time and Cost Burden
Source: Cancer Med. 2026 Feb 20;15(3):e71657. doi: 10.1002/cam4.71657 (PMC12928049; doi:10.1002/cam4.71657)

# Supplementary documents

**Table S1 Search strategy**

| Concept 1: Cancer | Search location |
| --- | --- |
| Cancer* OR oncology OR neoplasm OR tumor* OR carcinoma OR lymphoma OR sarcoma OR melanoma | Title, abstract, and subject heading |
| Concept 2: Informal caregiving |  |
| “informal care*” OR ‘informal caregiver’ OR ‘patient support’ OR ‘home care’ OR ‘unpaid care’ OR ‘informal support’ OR ‘family support’ OR ‘carers’ OR “supporter*” OR caregiver OR ‘carer family’ OR ‘partner’ OR ‘carer home’ OR caregive* OR family carer OR home carer OR Informal care* OR Informal caregive* OR unpaid care* | Title and abstract |
| Concept 3: time and cost |  |
| “time" OR hour* OR "period" OR "duration" OR cost* OR expense* OR “burden” | Title and abstract |
| Concept 1 AND Concept 2 AND Concept 3 |  |
| Limitation. Since 2015, peer-reviewed and English | |

**Table S2 Mean proportion of female caregivers, informal care hours, unit cost, and informal care cost of included studies**

| ID | Study | Proportion of female caregivers | Informal care hours (averaged within study and weekly costs calculated) | Unit cost PGA (averaged within studies) $2024 | Average unit cost OCA (averaged within studies) $2024 | Average monthly informal care cost  (averaged within study and monthly costs calculated) $2024 |
| --- | --- | --- | --- | --- | --- | --- |
| **High SDI** | | | | | | |
| 1 | Bayen | NA | 81.9 | NA | NA | 1649^3^ |
| 2 | Cai J. *et al* | 85 | 54·6 | NA | NA | NA |
| 3 | Chua C K T*. et al* | 56·3 | 84 | NA | NA | NA |
| 4 | Gridelli C*. et al* | 74 | NA | 31·67^1^ | NA | 1375^3,4^ |
| 5 | Halia O. *et al.* | NA | 11 | 27·07 | NA | 1429^5^ |
| 6 | Hanly P. *et al* a | 81·8 | NA | NA | 31·03 | 5626^3,6^ |
| 7 | Hanly P. *et al* b | 81·8 | 29.7 | 22·5^2^ | 29·6 | 2391^3,6^ |
| 8 | Hanly P*. et al* | NA | 17.8 | 20^2^ | 30·5 | 1824^3,7^ |
| 9 | Hao S. *et al* | NA | 17.5 | NA | 37 | No reported mean patient cost |
| 10 | Hendricks B.A *et al* | 82.7 | 22.43 | NA | NA | NA |
| 11 | Li c. *et al* | 100 | 1.3 | NA | NA | 748^7^ |
| 12 | Palandri | NA | NA | 985^1^ | NA | 3122^7^ |
| 13 | Roine E. *el al* | NA | 2·95 | 27·07 | NA | 346^3,8^ |
| 14 | Rowland C. *et al* | 64·2 | 7·92 | NA | NA | NA |
| 15 | Spatuzzi R. *et al* | 78 | 168 | NA | NA | NA |
| 16 | Stoffel T S. *et al* | NA | 15·8 | NA | NA | 348 |
| 17 | Tsimicalis A. *et al* | NA | 113.8 | NA | 49·8^1^ | 5327^3,4^ |
| 18 | van Houtven C H*. et al* | NA | 16 | NA | 26^1^ | 1913^7,9^ |
| 19 | van Houtven C.H*. et al* | 74 | 71.5 | NA | NA | NA |
| 20 | Wood R. *et al France* | NA | 24·2 | NA | NA | NA |
| 20 | Wood R. *et al Germany* | NA | 10·8 | NA | NA | NA |
| 20 | Wood R. *et al Italy* | NA | 40·1 | NA | NA | NA |
| 21 | Yabroff K R. *et al* | NA | 58·1 | NA | 27 | 3141^10^ |
| 22 | Hutchinson B. et al Australia | NA | 17·6 | NA | 15·5 | No reported mean patient cost |
| 22 | Hutchinson B. et al Canada | NA | 17·6 | NA | 12·2 | No reported mean patient cost |
| 22 | Hutchinson B. et al UK | NA | 17·6 | NA | 12·4 | No reported mean patient cost |
| 22 | Hutchinson B. et al USA | NA | 17·6 | NA | 8 | No reported mean patient cost |
| 23 | Luengo‑Fernandez et al Austria | NA | NA | NA | 19^1^ | No reported mean patient cost |
| 23 | Luengo‑Fernandez et al Belgium | NA | NA | NA | 22^1^ | No reported mean patient cost |
| 23 | Luengo‑Fernandez et al Cyprus | NA | NA | NA | 14^1^ | No reported mean patient cost |
| 23 | Luengo‑Fernandez et al Czech Republic | NA | NA | NA | 6^1^ | No reported mean patient cost |
| 23 | Luengo‑Fernandez et al Denmark | NA | NA | NA | 29^1^ | No reported mean patient cost |
| 23 | Luengo‑Fernandez et al Estonia | NA | NA | NA | 5^1^ | No reported mean patient cost |
| 23 | Luengo‑Fernandez et al Finland | NA | NA | NA | 22^1^ | No reported mean patient cost |
| 23 | Luengo‑Fernandez et al France | NA | NA | NA | 18^1^ | No reported mean patient cost |
| 23 | Luengo‑Fernandez et al Germany | NA | NA | NA | 24^1^ | No reported mean patient cost |
| 23 | Luengo‑Fernandez et al Greece | NA | NA | NA | 15^1^ | No reported mean patient cost |
| 23 | Luengo‑Fernandez et al Ireland | NA | NA | NA | 23^1^ | No reported mean patient cost |
| 23 | Luengo‑Fernandez et al Latvia | NA | NA | NA | 4^1^ | No reported mean patient cost |
| 23 | Luengo‑Fernandez et al Lithuania | NA | NA | NA | 4^1^ | No reported mean patient cost |
| 23 | Luengo‑Fernandez et al Luxembourg | NA | NA | NA | 29^1^ | No reported mean patient cost |
| 23 | Luengo‑Fernandez et al Netherlands | NA | NA | NA | 22^1^ | No reported mean patient cost |
| 23 | Luengo‑Fernandez et al Poland | NA | NA | NA | 5^1^ | No reported mean patient cost |
| 23 | Luengo‑Fernandez et al Slovenia | NA | NA | NA | 9^1^ | No reported mean patient cost |
| 23 | Luengo‑Fernandez et al Spain | NA | NA | NA | 13^1^ | No reported mean patient cost |
| 23 | Luengo‑Fernandez et al Sweden | NA | NA | NA | 22^1^ | No reported mean patient cost |
| 23 | Luengo‑Fernandez et al UK | NA | NA | NA | 17^1^ | No reported mean patient cost |
|  | **Mean** | **78** | **38.3** | **23** | **19** | **2,249** |
| **High middle SDI** | | | | | | |
| 24 | Yang Y. *et al* | NA | 63 | NA |  | 85^11^ |
| 25 | Cicin I. *et al* | NA | NA | NA | NA | 39,056,884 for 47,500 persons or 69 per person per month |
| 22 | Hutchinson B*. et al* Kazakhstan | NA | NA | NA | 0.8 | No reported mean patient cost |
| 22 | Hutchinson B*. et al* Malaysia | NA | NA | NA | 2.2 | No reported mean patient cost |
| 23 | Luengo‑Fernandez 2013 Bulgaria | NA | NA | NA | 2^1^ | No reported mean patient cost |
| 23 | Luengo‑Fernandez 2013 Hungary | NA | NA | NA | 5^1^ | No reported mean patient cost |
| 23 | Luengo‑Fernandez 2013 Italy | NA | NA | NA | 16^1^ | No reported mean patient cost |
| 23 | Luengo‑Fernandez 2013 Malta | NA | NA | NA | 9^1^ | No reported mean patient cost |
| 23 | Luengo‑Fernandez 2013 Portugal | NA | NA | NA | 9^1^ | No reported mean patient cost |
| 23 | Luengo‑Fernandez 2013 Romania | NA | NA | NA | 3^1^ | No reported mean patient cost |
| 23 | Luengo‑Fernandez 2013 Slovakia | NA | NA | NA | 5^1^ | No reported mean patient cost |
|  | **Mean** |  | **32**·**7** |  | **6** | **77** |
| **Middle SDI** | | | | | | |
| 22 | Hutchinson B*. et al* Colombia | NA | 17·6 | NA | 1·5 | No reported mean patient cost |
| **Low Middle SDI** | | | | | | |
| 22 | Hutchinson B*. et al* India | NA | 17·6 | NA | 0·4 | No reported mean patient cost |
| 22 | Hutchinson B*. et al* Kenya | NA | 17·6 | NA | 0·9 | No reported mean patient cost |
| 22 | Hutchinson B*. et al* Nigeria | NA | 17·6 | NA | 0·4 | No reported mean patient cost |
| 26 | Eze N C *et al* | 62^.^9 | 104^.^4 |  |  |  |
|  | **Mean** | **62^.^9** | **39.3** |  | **0**·**6** |  |
| **Low SDI** | | | | | | |
| 22 | Hutchinson B*. et al* Malawi | NA | 17·6 | NA | 2·1 | No reported mean patient cost |

*Note: 1. Average of all unit cost was considered 2. Only the first reported unit cost was included, this study used various other unit costs 3. Average of costs included 4. 3 months cost was divided by 3 to calculate monthly cost 5. All cancers costs were included, and monthly cost was calculated by dividing by caregiving duration (in days) and multiplying by 30 6. Weekly cost multiplied by 4 to calculate monthly cost 7. Yearly cost divided by 12 to calculate monthly cost 8. Costs over 6 months divided by 6 to calculate monthly cost 9. Average of full sample of caregiver’s costs was included 10. All cancers included and cost over 2 years was divided by 24 to calculate monthly costs 11. Monthly cost was calculated by dividing with the cost since diagnosis in months*

*When the study among children (Tsimicalis et al) was removed, informal care hours per week in high SDI countries was 33.90 per week and the average unit cost (OCA) was US$18 per hour.*

*NA: Not available OCA: Opportunity cost approach; PGA: Proxy good approach SDI: Socio demographic index*

*For informal care hours: *Note: Weekly hours calculation method examples:*

1. *Daily hours were multiplied by 7 to calculate weekly hours (Bayen et al 11.7*7=81.9) 2. Median hours were used* Ex Cai et al: 115.14+103.13)/2 *3. Hours over 2 weeks was divided by 2 to calculate weekly hours Cai et al: (*(115.14+103.13)/2))/2 *4. Data for highest proportion of respondents was considered (Chua ey al 12 hrs*7) 5. Average of all phases/patient types was calculated (Hanly et al 2013 b((42.5+16.9)/2) 6. Data over 3 months was divided by 12 to calculate weekly data 7. Data for overall was used 8. Data for mother and father was averaged*

**Table S3: Results of risk of bias analysis**

**Cost of Illness studies**

|  |  | Hao S. *et al* | Tsimicalis A. *et al* | Circin I. *et al* | Hutchinson B*. et al* | Luengo‑Fernandez |
| --- | --- | --- | --- | --- | --- | --- |
| **Study characteristics** | Question/objective | Yes | Yes | Yes | Yes | Yes |
|  | Population | Yes | Yes | Yes | Yes | Yes |
|  | Perspective | Yes | Yes | Yes | Yes | Yes |
| **Methodology and cost analysis** | Epidemiological approach | Yes | Unclear | Unclear | Yes | Yes |
|  | Costing approach | Yes | Yes | Yes | Yes | Yes |
|  | Data collection approach | Yes | Yes | Unclear | Unclear | Unclear |
|  | Identification | Yes | Yes | Yes | Yes | Yes |
|  | Measurement | Yes | Yes | Yes | Yes | Yes |
|  | Valuation | Yes | Yes | Yes | Yes | Yes |
|  | Time horizon | Yes | Yes | Yes | Yes | Yes |
|  | Discounting | NA | NA | NA | Yes | Yes |
|  | Sensitivity | Yes | Yes | No | No | Yes |
| **Results and reporting** | Cost sectors | Yes | Yes | Yes | Yes | Yes |
|  | Generalizability | Yes | Yes | yes | yes | yes |
|  | Limitations | Yes | Yes | Yes | Yes | Yes |
|  | Ethical and distributional issues | Yes | Yes | Yes | Yes | Yes |
|  | Conflict of interest | No | No | No | Yes | Yes |

**Cross sectional studies**

|  | Bayen *et al* | Chua C K T*. et al* | H-Hanly P. *et al* 2013 a | T-Hanly P. *et al* 2013b | M-Hanly P*. et al 2017* | Li c. *et al* | Palandri *et al* | Roine E. *el al* |
| --- | --- | --- | --- | --- | --- | --- | --- | --- |
| Were the criteria for inclusion in the sample clearly defined? | Yes | Yes | Yes | Yes | Yes | Yes | Yes | Yes |
| Were the study subjects and the setting described in detail? | Yes | Yes | Yes | Yes | Yes | Yes | Yes | Yes |
| Was the exposure measured in a valid and reliable way? | Yes | Yes | Yes | Yes | Yes | Yes | Yes | Yes |
| Were objective, standard criteria used for measurement of the condition? | Yes | Yes | Yes | Yes | Yes | Yes | Yes | Yes |
| Were confounding factors identified? | No | No | No | No | No | No | No | No |
| Were strategies to deal with confounding factors stated? | NA | NA | NA | NA | NA | NA | NA | NA |
| Were the outcomes measured in a valid and reliable way? | Yes | Yes | Yes | Yes | Yes | Yes | Yes | Yes |
| Was appropriate statistical analysis used? | Yes | Yes | Yes | Yes | Yes | Yes | Yes | Yes |

|  | Rowland C. *et al* | Spatuzzi R. *et al* | Stoffel T S. *et al* | van Houtven C H V*. et al* | Wood R. *et al* | Yabroff K R. *et al* | Yang Y. *et al* |
| --- | --- | --- | --- | --- | --- | --- | --- |
| Were the criteria for inclusion in the sample clearly defined? | Yes | Yes | Yes | Yes | Yes | Yes | Yes |
| Were the study subjects and the setting described in detail? | Yes | Yes | Yes | Yes | Yes | Yes | Yes |
| Was the exposure measured in a valid and reliable way? | Yes | Yes | Yes | Yes | Yes | Yes | Yes |
| Were objective, standard criteria used for measurement of the condition? | Yes | Yes | Yes | Yes | Yes | Yes | Yes |
| Were confounding factors identified? | No | No | No | No | No | No | No |
| Were strategies to deal with confounding factors stated? | NA | NA | NA | NA | NA | NA | NA |
| Were the outcomes measured in a valid and reliable way? | Yes | Yes | Yes | Yes | Yes | Yes | Yes |
| Was appropriate statistical analysis used? | Yes | Yes | Yes | Yes | Yes | Yes | Yes |

|  | Rowland C. *et al* | Spatuzzi R. *et al* | Stoffel T S. *et al* | van Houtven C H V*. et al* | Wood R. *et al* | Yabroff K R. *et al* | Yang Y. *et al* |
| --- | --- | --- | --- | --- | --- | --- | --- |
| Were the criteria for inclusion in the sample clearly defined? | Yes | Yes | Yes | Yes | Yes | Yes | Yes |
| Were the study subjects and the setting described in detail? | Yes | Yes | Yes | Yes | Yes | Yes | Yes |
| Was the exposure measured in a valid and reliable way? | Yes | Yes | Yes | Yes | Yes | Yes | Yes |
| Were objective, standard criteria used for measurement of the condition? | Yes | Yes | Yes | Yes | Yes | Yes | Yes |
| Were confounding factors identified? | No | No | No | No | No | No | No |
| Were strategies to deal with confounding factors stated? | NA | NA | NA | NA | NA | NA | NA |
| Were the outcomes measured in a valid and reliable way? | Yes | Yes | Yes | Yes | Yes | Yes | Yes |
| Was appropriate statistical analysis used? | Yes | Yes | Yes | Yes | Yes | Yes | Yes |

|  | Eze N. C et al | Hendricks B. A. et al | van Houtven C. H. et al 2025 |
| --- | --- | --- | --- |
| Were the criteria for inclusion in the sample clearly defined? | Yes | Yes | Yes |
| Were the study subjects and the setting described in detail? | Yes | Yes | Yes |
| Was the exposure measured in a valid and reliable way? | Yes | Yes | Yes |
| Were objective, standard criteria used for measurement of the condition? | Yes | Yes | Yes |
| Were confounding factors identified? | No | No | No |
| Were strategies to deal with confounding factors stated? | NA | NA | NA |
| Were the outcomes measured in a valid and reliable way? | Yes | Yes | Yes |
| Was appropriate statistical analysis used? | Yes | Yes | Yes |

**Cohort studies**

|  | Cai J. *et al* | Gridelli C*. et al* | Haltia O. *et al.* |
| --- | --- | --- | --- |
| Were the two groups similar and recruited from the same population? | NA | NA | NA |
| Were the exposures measured similarly to assign people to both exposed and unexposed groups? | NA | NA | NA |
| Was the exposure measured in a valid and reliable way? | NA | NA | NA |
| Were confounding factors identified? | No | No | No |
| Were strategies to deal with confounding factors stated? | No | No | No |
| Were the groups/participants free of the outcome at the start of the study (or at the moment of exposure)? | No | No | No |
| Were the outcomes measured in a valid and reliable way? | Yes | Yes | Yes |
| Was the follow up time reported and sufficient to be long enough for outcomes to occur? | Yes | Yes | Yes |
| Was follow up complete, and if not, were the reasons to loss to follow up described and explored? | Yes | Yes | Yes |
| Were strategies to address incomplete follow up utilized? | Yes | Yes | Yes |
| Was appropriate statistical analysis used? | Yes | Yes | Yes |

**Table S4 Data for meta-analysis**

| **First Author** | **Sample size** | **Caregiving hours per day** | **Caregiving hours per week unless specified** | **Calculated hours per week** | **SD** | **SE** | **LCI** | **UCI** | **Assumption** |
| --- | --- | --- | --- | --- | --- | --- | --- | --- | --- |
| Li c. *et al 2013* | 88 | NA | 1.3 | 1.3 | 3.3 | 0.14 | 1.00 | 1.53 | Mean informal caregiving hours(SD) is 65.9(172.4) over 1 year after diagnosis in Table 4 |
| Yabroff K R. *et al 2009* | 688 | NA | NA | 58.1 | 58.1 | 2.22 | 53.76 | 62.44 | For sample size, this is the number of patients, and so we assume 1:1 patient and caregiver ratio |
| Hanly P*. et al 2017* | 583 | NA | 17.8 | 17.8 | NA | 0.74 | 16.36 | 19.24 | None |
| Rowland C. *et al 2017* | 1504 | 13.6 | 95 for 3 months | 7.91 | 10.3 | 0.20 | 7.52 | 8.32 | Assuming 4 weeks in a month and the duration is 3 months |
| Tsimicalis A. et al | 254 | NA | 1365 over 3 months | 113.8 | 48.3 | 7.14 | 99.76 | 127.74 | Divided by 12 as 12 weeks in 3 months |
| Hanly 2013b | 154 | NA | 16.9 | 16.9 | 20.1 | 1.36 | 14.23 | 19.57 | Considered  Ongoing care phase |
| Eze2025 | 105.0 | 14.92 | NA | 104.4 | 57.12 | 10.19 | 84.46 | 124.42 |  |
| Van Houtven2025 | 198 | 10.21 | NA | 71.47 | 49.77 | 5.08 | 61.51 | 81.43 |  |

*LCI: Lower confidence interval; SD: Standard deviation; SE: Standard error; UCI: Upper confidence interval*

**Table S5 Forest plot of meta-analysis of informal care hours per week**

**S5.1 Meta-analysis of all studies (first and second screening round)**

**S5.2 Meta-analysis of of all studies (first and second screening round) after removing Tsimicalis 2012**

**S5.3 Meta-analysis of studies of the first screening round**

*Note: The Study column included author, year of publication and the risk of bias scores (number of parameters scored as yes/total number of parameters in the scoring method)*

A total of six studies reported the mean, sample size, SD/95% CI and all were from high SDI. The pooled informal care hours per week was 35.60 (95% CI: 1.67-69.53) with a high heterogeneity (I^2^=100%).

**S5.4 Meta-analysis of studies of first screening round after removing Tsimicalis 2012**

Meta-analysis after removing Tsimicalis 2012, which was conducted on children led to the reduction in the informal care hours to 20.33 per week.


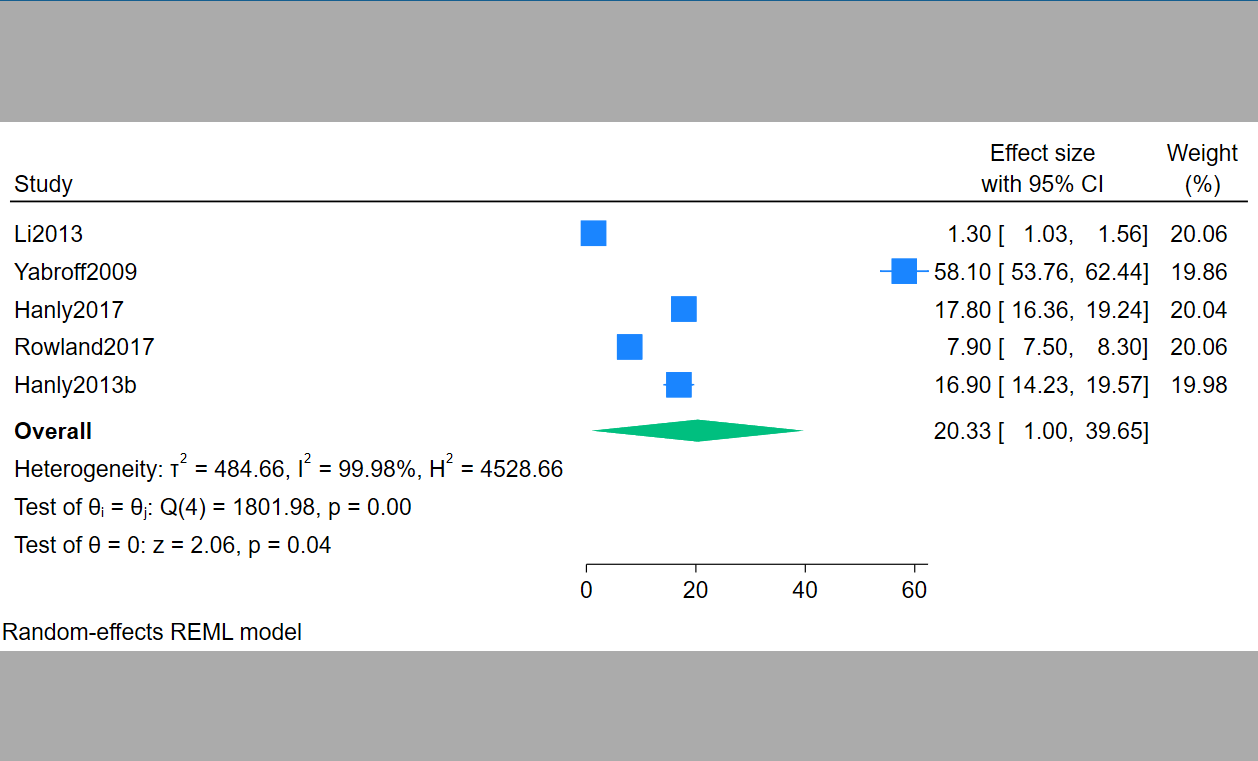

Supplement: Supplementary file 1 — Table S1: cam471657‐sup‐0001‐TablesS1‐S5.docx. [file CAM4-15-e71657-s001.docx]
